# Expanding the knowledge translation metaphor

**DOI:** 10.1186/s12961-017-0184-x

**Published:** 2017-03-13

**Authors:** Eivind Engebretsen, Tony Joakim Sandset, John Ødemark

**Affiliations:** 1 Institute of Health and Society, Faculty of Medicine, University of Oslo, BOX 1130, Blindern, 0318 Oslo Norway; 2 Department of Cultural Studies and Oriental Languages, Faculty of Humanities, University of Oslo, BOX 1010, Blindern, 0315 Oslo Norway

**Keywords:** Knowledge translation, Evidence-based medicine, Humanities, Social science

## Abstract

**Background:**

Knowledge translation (KT) is a buzzword in modern medical science. However, there has been little theoretical reflection on translation as a process of meaning production in KT. In this paper, we argue that KT will benefit from the incorporation of a more theoretical notion of translation as an entangled material, textual and cultural process.

**Discussion:**

We discuss and challenge fundamental assumptions in KT, drawing on theories of translation from the human sciences. We show that the current construal of KT as separate from and secondary to the original scientific message is close to the now deeply compromised literary view of translation as the simple act of copying the original. Inspired by recent theories of translation, we claim that KT can be more adequately understood in terms of a ‘double supplement’ – on the one hand, KT offers new approaches to the communication of scientific knowledge to different groups in the healthcare system with the aim of supplementing a lack of knowledge among clinicians (and patients). On the other, it demonstrates that a textual and cultural supplement, namely a concern with target audiences (clinicians and patients), is inevitable in the creation of an ‘autonomous’ science. Hence, the division between science and its translation is unproductive and impossible to maintain. We discuss some possible implications of our suggested shift in concept by drawing on pharmaceutical interventions for the prevention of HIV as a case. We argue that such interventions are based on a supplementary and paradoxical relation to the target audiences, both presupposing and denying their existence.

**Summary:**

More sophisticated theories of translation can lay the foundation for an expanded model of KT that incorporates a more adequate and reflective description of the interdependency of scientific, cultural, textual and material practices.

## Background

Several articles in *BMC* journals have recently drawn attention to fundamental concepts within evidence-based medicine and knowledge translation (KT) [1–4]. Greenhalgh et al. [3] have questioned the dominant notion of evidence in evidence-based medicine by drawing attention to six biases’ against patients and care givers. Kelly et al. [1] have emphasised the often underestimated role of values in evidence-based decision making. More recently, Greenhalgh et al. [4] have explored the notion of research impact and its philosophical basis. We will add to this debate by challenging and expanding the metaphor of KT; we are aware that we are not the first to challenge this metaphor [5]. However, while other authors have mainly questioned the knowledge aspect of the concept, we are more interested in what the term ‘translation’ has to offer and how it might be expanded.

The term ‘translation’ has become increasingly important in the contemporary natural and human sciences. On the one hand, the turn to translation can be traced across a number of human sciences, such as cultural history, anthropology, and science and technology studies [6]. On the other hand, translation has lately become institutionalised in the field of medicine, leading to the development of ‘knowledge translation’ and ‘translational research’. These concepts refer to a set of research activities bound together by the common goal of ‘bridging the gap’ between laboratory science and clinical application, and more generally, putting research-based knowledge into practice [5, 7–9]. While translation in the human sciences has emerged as a key theoretical concept, which has problematised the complex interfaces of textual, cultural and material transgression and exchange, its materialisation in medical discourse is of an entirely different nature. In medicine, KT denotes a scientific and purportedly non-cultural practice that defines social and cultural differences as a ‘barrier’ to the transmission of medical science. The aim of KT is to bring pure scientific knowledge from ‘bench to bedside’ by testing its validity in clinical practice, while at the same time keeping the scientific knowledge intact throughout the process of translation across various social fields and sectors of the healthcare system.

With few exceptions [5, 10], however, there has been little theoretical reflection on translation as a process of meaning production in KT. In this paper, we first argue that KT is based on a simplistic view of translation and knowledge dissemination, a view that to a large extent takes translation as a phenomenon for granted. Second, we maintain that the practice of KT might benefit from incorporating more theoretical notions of translation as an entangled material, textual and cultural process which inevitably impacts the ‘original scientific message’. The fact that translation has become a commonplace (*topos*) in modern science with the ability to assemble an array of divergent approaches and practices under one name makes it a key instrument for transdisciplinary translation and exchange. In this situation, concepts and practices of translation have an unexploited potential for bridging the gap between medicine and social/human sciences. Such interdisciplinary exchange can, in turn, contribute to an increased understanding of the interplay between scientific and cultural factors of KT and thereby ultimately enhance the flow of knowledge within healthcare. Hence, rather than dropping the KT metaphor, we should extend it by taking advantage of discourses and practices of translation in the humanities.

## Discussion

### Translation in medicine

KT has generally been conceptualised in terms of a chain involving distinct stages of production and dissemination [9]. The most common current model comprises three stages of translation, namely T1, a passage from basic laboratory science to clinical research on populations (aka translational research); T2, from clinical research to clinical recommendations, often in terms of the development of clinical guidelines based on systematic reviews of clinical trials; and T3, from clinical recommendations to routine clinical practice [7].

Here, translation is conceived of as a process of testing and synthetising scientific knowledge produced in the laboratory to prepare it for sound clinical application and scientifically warranted healthcare [11]. The underlying assumption is that translation – if it is to be felicitous –is non-productive. Its principal purpose is to preserve and implement the original, scientific content in new sociocultural contexts (practical healthcare in individual cases across the globe). The influence of the ‘target culture’ (clinical practice) on the original (scientific) message should be limited as far as possible. The translational act itself is a non-act and the translator a non-actor; the purpose of translation is to be a ‘container’ of the original message without adding, transforming or otherwise ‘betraying’ the original.

This presupposes that it is possible to separate the production of knowledge from its transfer; the scientific content to be translated is construed as being outside the process of translation. The same distinction between production and transfer is also inherent in the dominant definition of KT as “*exchange, synthesis and ethically-sound application of knowledge*” [8]. This definition reduces the act of translation to activities (exchange, synthesis and application) that are structurally and temporally separate from the production of knowledge. In line with this, so-called barriers and drivers of KT are essentially understood as social and cultural factors external to the production of knowledge [12]. Knowledge, moreover, is said to have reached its culmination in the ‘secluded space’ of controlled trials, and it is the results from these trials that should be transported to, and implemented in, practical care situations. To accomplish this, various textual genres are mobilised in the different stages of the translation process, culminating in so-called clinical guidelines, which prescribe manners of intervention in concrete cases (e.g. particular diagnoses, prognoses or treatments) based on systematic reviews of the scientific state of the art. Hence, the translation process hinges upon textualisation in such genres as systematic reviews and guidelines, and that such texts, in increasingly condensed and vernacular forms, are able to transmit the science necessary to implement state-of-the-art care. The transference of the message from one textual genre to another should not modify the scientific content.

### Translation as a textual and cultural supplement

The current construal of KT is actually close to the now deeply compromised literary view of translation as a practice that aims at creating a semantic or pragmatic equivalence between an original ‘source text’ and a new ‘target text’ [13], a process governed by the norm of fidelity to the source, and in which the translator’s work is ‘invisible’ [14] and merely ‘ancillary’ [15]. As emblematically formulated by Nabokov [16], “*the person who desires to turn a literary masterpiece into another language has only one duty to perform, and this is to reproduce with absolute exactitude the whole text, and nothing but the text*”. However, more recent scholars in translation studies have emphasised that the original source text can never be fully recovered by the target text/culture, that translations always imply semantic shifts, and must be “*rewritten in domestic dialects and discourses, registers and styles*” [17]. Besides, the importance of cultural factors has been underscored. Lefevere [18] has, for instance, maintained that problems in translation are not primarily of a linguistic nature. Rather, questions of translatability have more to do with cultural factors, what he refers to as “*discrepancies in the conceptual and textual grids*”, than with “*discrepancies in languages*” [18]. Interpreting the phrase ‘once upon a time’ as different from ‘a long time ago’, for instance, requires knowledge of cultural genres. Such cultural and textual framing cannot be read out of the sentence as mere linguistic data. Linguistic translation, then, also has to account for cultural factors, such as metadiscursive framings/practices, and different styles of reasoning [19].

According to Derrida [20], translation is an integral part of all textual production; the translation or target text relates to the source text as what Derrida has referred to as a “*double supplement*” – it both adds on to the original and compensates for a lack in the original. The translation does not only duplicate the original message, it also completes the original message (‘the supplement supplements’) by fulfilling one of its possible interpretations. If, then, shifts of meaning are an inevitable outcome of the transport of signs between texts and cultures, KT could become more effective if such shifts were defined as a creative potential rather than as a ‘barrier’.

We maintain that KT relates to the ‘original scientific content’ as a double supplement. On the one hand, KT offers new approaches to the communication of scientific knowledge to different groups in the healthcare system with the aim of supplementing a lack of knowledge among clinicians (and patients). On the other, it demonstrates that a textual and cultural supplement, namely a concern with target audiences (clinicians and patients), is inevitable in the creation of an ‘autonomous’ science. This creates an inherent paradox in existing KT models – while these models presuppose that the principal duty of adequate KT is to implement the original scientific message in new social contexts and textual forms without altering its content, the same models, paradoxically, also state that it is through translational modifications and adaption to new audiences, i.e. through synthesis and development of guideline recommendations, that the message becomes scientifically trustworthy. Hence, translation both threatens and fulfils the original scientific message. However, existing KT models fail to draw the consequences from this paradox – translation is inherent in science and the division between science and its translation is both impossible and unproductive to maintain.

### Practical implications: the case of pre-exposure prophylaxis (PrEP)

An illustrative example of this interdependency of science and translation is the knowledge development within HIV prevention. PrEP challenges the mere focus on sexual behaviour change in HIV prevention, which is considered to have been inadequate by most of the advocates of the biomedicalisation of HIV prevention [21]. The principal preventive approaches developed over the course of the first period in the fight against AIDS were based on attempts to change behaviours, with the establishment of the social norm of safe sex and condom use as a means of protection against HIV. The PrEP paradigm is grounded on a different logic – bringing medication to the maximum number of people infected with HIV will not only bring the promise of greatly enhanced survival and quality of life for people living with HIV, but will also greatly reduce their viral loads and the likelihood of passing the virus onto new people [22]. The effectiveness of PrEP is backed by randomised controlled trials (RCTs) demonstrating that medication provides protection against the acquisition of HIV infection, if the drug is taken regularly.

However, through their study design, the RCTs in question control the social behaviour of the participants, notably by including frequent HIV counselling and testing during enrolment. In addition, behavioural interventions (e.g. individualised motivational interviewing, risk-reduction counselling) are used to assist participants in overcoming obstacles to pill use [23]. The problem with these studies is that they try to distinguish between the scientific message and its translation to the target culture while at the same time demonstrating their interdependency; on the one hand, the RCT secludes the intervention in a ‘controlled world’ independent of the messiness of the social behaviour context that will necessarily affect its translation into practice. On the other hand, it is the same messiness of the social behaviour context that motivates the whole intervention; sexual behaviour interventions are considered insufficient due to a lack of adherence in the target culture. The RCT preconfigures an ‘ideal user’ who is taking the medication regularly, a user that the same studies (by introducing PrEP as a pharmaceutical supplement) assume to be non-existent. The effect of the drug is tested independently of the social dimensions that motivated the intervention in the first place. Here, a paradox arises, namely if sexual behaviour interventions are now regarded as inadequate – due to their reliance on a human agent who is supposed to make right choices in messy social contexts – PrEP actually assumes the very same kind of agency and the ability of rationally choosing in order to follow the PrEP regime.

Our point is that scientific evidence cannot be obtained without acknowledging the active contribution of particular target cultures and the creative potential of KT. Understanding KT as a cultural and textual supplement, as we suggest, is to acknowledge that altering the scientific message is a necessary and integral part of KT. Modifications and changes that occur through the translational process should therefore not be viewed as ‘barriers’ to accurate translation; rather such changes are prerequisites for evidence-based healthcare.

## Conclusion

The linear conception of translation inherent in KT has motivated researchers to abandon the metaphor and replace it with notions such as ‘co-creation’ or ‘transformation’ [5]. We instead advocate that there is a theoretical and practical potential in the concept of translation that risks being lost with the introduction of a new terminology. By searching to conceptualise the balance between fidelity and creativity, between (scientific) content and culture, and between technical and political considerations, modern theories of translation can serve as an ‘epistemological lubricant’ [6] facilitating the transfer of knowledge within healthcare, but also between medicine and the human sciences. Such theories can lay the foundation for an expanded model of KT that incorporates a more dynamic conception of translation and a more adequate and reflective description of the interdependency of scientific, cultural, textual and material practices. This can, in turn, facilitate a better integration of research evidence and knowledge developed within the messy domain of ‘practice’, including both patient/user knowledge and clinical expertise.
